# Flow reserve fraction: the optimal choice in lesion assessing and interventional guiding for patient with unstable angina pectoris and intermediate lesion wrapped with myocardial bridge: a case report

**DOI:** 10.1186/s13019-021-01720-7

**Published:** 2021-11-21

**Authors:** Yuecheng Hu, Hongliang Cong, Liuying Zheng, Dongxia Jin

**Affiliations:** Department of Cardiology, Tianjin Chest Hospital, Tianjin University, Chest Clinical Medical College of Tianjin Medical University, No. 261, Taierzhuang South Road, Jinnan District, Tianjin, 300222 China

**Keywords:** Unstable angina pectoris, Flow reserve fraction, Intravenous ultrasound, Percutaneous coronary intervention, Myocardial bridge

## Abstract

**Background:**

It is difficult to choose correctly interventional strategy for coronary intermediate lesions combined with myocardial bridge. Endovascular imaging is advocated to guide treatment, but flow reserve fraction (FFR) is not recommended to guide the interventional treatment of myocardial bridge disease because of the inaccurate judgment misled by myocardial bridge.

**Case presentation:**

In this study, we reported a case of a 56-year-old male patient with unstable angina pectoris (UAP). From his coronary angiography, we found diffuse stenosis near the midsection of the left anterior descending (LAD) branch and the presence of a severe myocardial bridge in the lesion area. We were sure that the LAD was culprit vessel and this lesion was culprit lesion. Both FFR and intravenous ultrasound (IVUS) were performed and the conclusions of them are different. Although stent implantation is not usually recommended in the myocardial bridge area. However, after careful examination, a stent was finally implanted under the precise guidance of FFR. And the patient recovered well up-to now.

**Conclusions:**

This case illustrates that FFR functional test was complimentary to intravascular imaging test for the coronary intermediate lesion, especially the lesion wrapped with myocardial bridges, both in assessing the lesion and in guiding treatment**.**

## Highlights

1. It is very difficult to choose the appropriate treatment strategy for coronary artery lesions with myocardial bridge.
2. The functional evaluation represented by FFR is better than the imaging evaluation represented by IVUS for the evaluation of this kind of lesions intervention or not.
3. FFR is complimentary to IVUS in the choice of interventional therapy for this kind of lesions.

## Background

How to choose interventional treatment strategy for coronary artery stenosis combined with myocardial bridge has always been the focus of cardiologist. There is no consensus or guidelines in this field. Physicians of different clinical intervention centers have diverse views. Endovascular imaging is advocated by majority physicians to guide treatment, while functional test, such as FFR, is not recommended to guide the interventional treatment of myocardial bridge disease because of the inaccurate judgment misled by myocardial bridge. Anyhow we insist that functional test such as FFR is superior to endovascular imaging, because this case suggested that FFR can be a good evaluation of interventional strategy for coronary artery stenosis combined with myocardial bridge.

## Case presentation

A 56-year-old male patient was admitted to the hospital with "paroxysmal chest pain for 1 month". He had no bad habits such as smoking or drinking, no history of diabetes and hypertension. Electrocardiogram (ECG) showed ST segment depression of 0.2–0.4 mV in leads V2-V5. High-sensitive troponin T was normal. Cardiac echocardiography showed that LVEDD was 48 mm and LVEF was 60%. He was diagnosed with unstable angina pectoris.

**Fig. 1:**
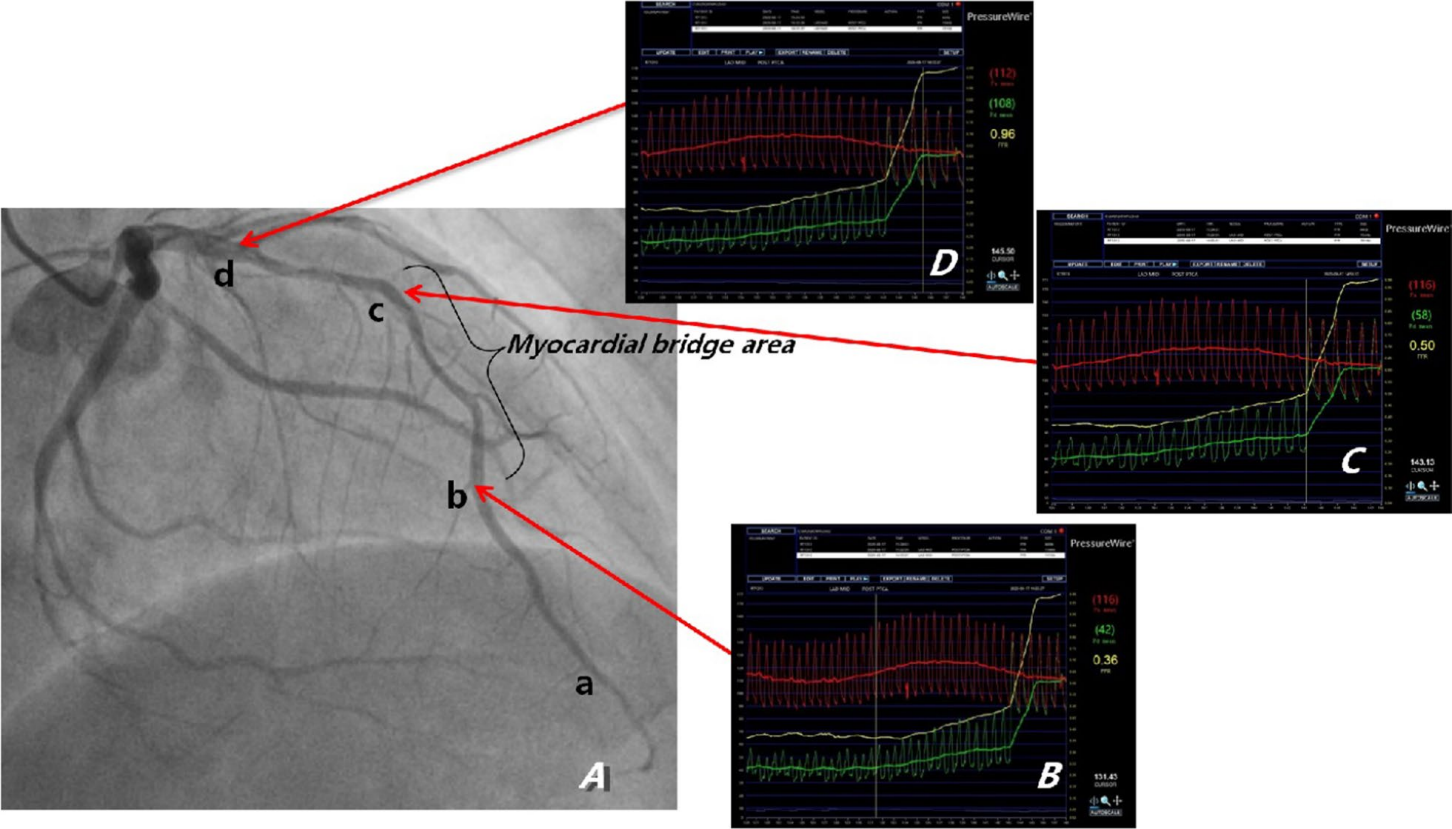
In the coronary angiography, a, b, c and d points were marked respectively from the distal to the proximal of LAD. The culprit lesion was located from b to d, which are wrapped with myocardial bridge. The coronary blood fractional flow reserve (FFR) was performed. After maximal congestion with adenosine, the pressure at point a and b were both 0.36 (normal value > 0.8), the pressure at point c was 0.50, and the pressure at point d was 0.96

**Fig. 2:**
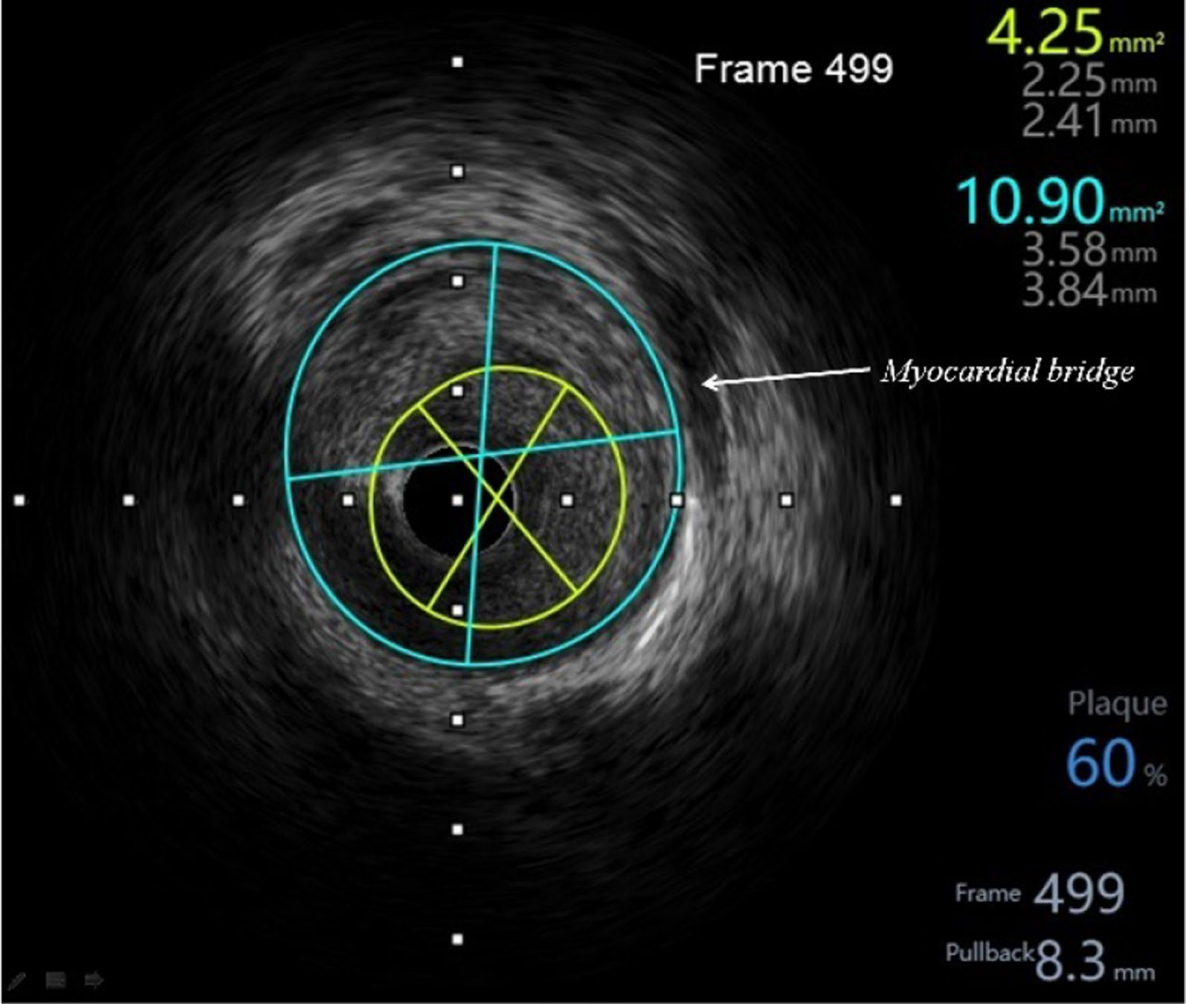
IVUS examination before PCI showed that the minimum lumen cross-sectional area was 4.25mm^2^, the plaque load was 60%, and there was a large amount of fiber and lipid components, but no plaque rupture and dissection. The arrow refers to the myocardial bridge

**Fig. 3:**
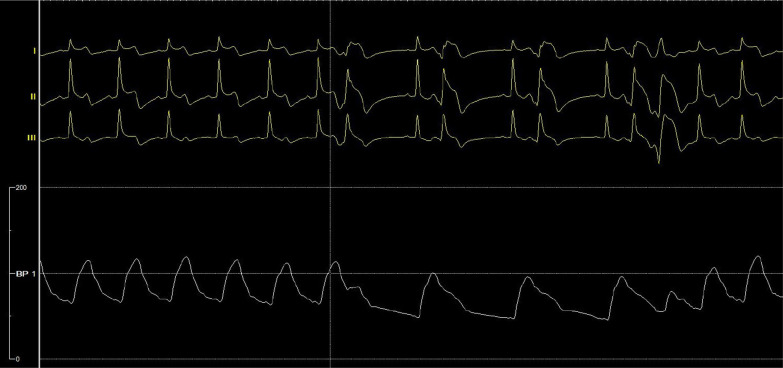
Intra-coronary injection of adenosine, ECG monitoring showed ST-segment elevation of 0.2–0.6 mV in II and III leads, and frequent premature ventricular beats

**Fig. 4:**
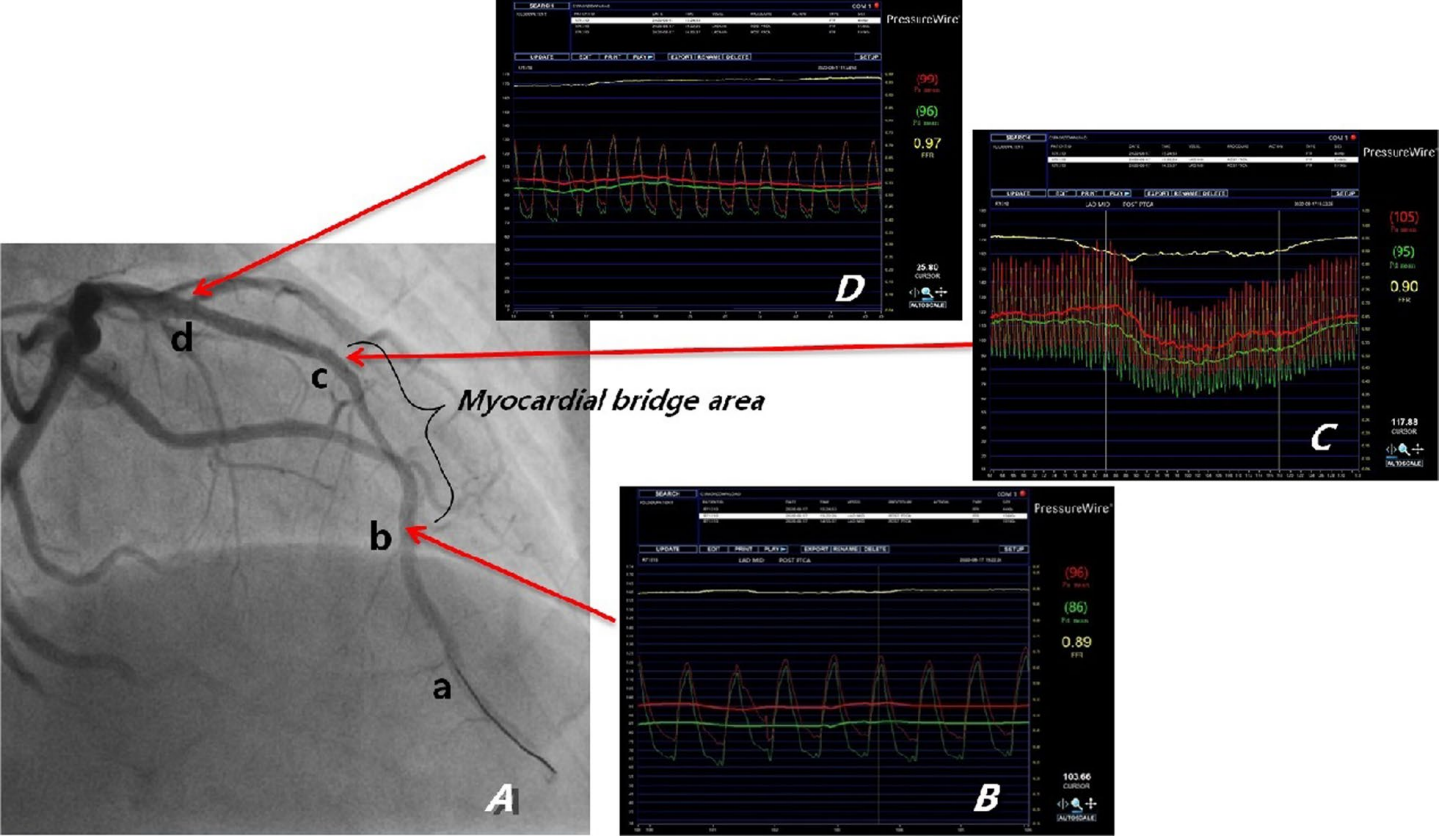
Angiography and FFR after stent implantation. A stent was implanted from C to D. FFR was performed again. After maximal congestion with adenosine, the values of a and b were both 0.89 (normal value > 0.8), and the pressure at point C is 0.90, and the pressure at point D is 0.96. The pressure gradient of C–D zone is 0.07, and the pressure gradient of B–C zone is 0.01

**Fig. 5:**
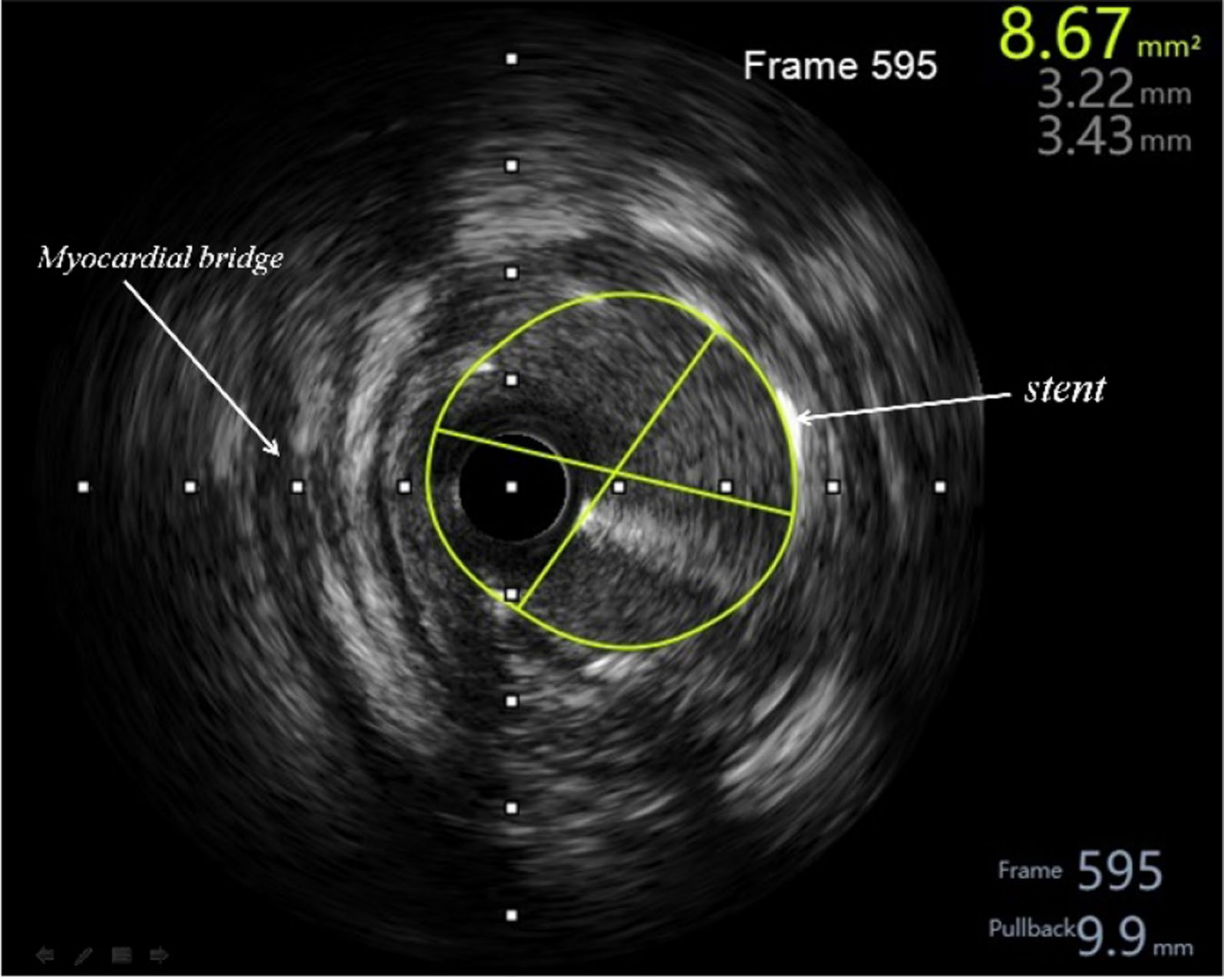
IVUS examination after PCI showed that the minimum area of the stent is 8.67mm^2^, with good sticking and expansion, and the stent was well expanded and apposed, without coronary dissection at both ends of the stent. The arrow refers to the myocardial bridge

After admission, he was given conventional drug treatment, and coronary angiography was performed the next day. It showed that the culprit lesion was in the middle segment of LAD with it’s lumen stenosis of 60–70%, which was intermediate but diffuse, and what’s more there was a moderate to severe myocardial bridge in the lesion segment (Fig. 1a). And there was no lesions in right coronary and left circumflex artery. Then intravascular ultrasound was performed with the results: minimum lumen cross-sectional area (MLA) of the lesion was 4.25mm^2^, with the length of 44 mm, and plaque load was 60%, and the plaque was made of plenty of fiber and lipid composition, without plaque rupture and dissection (Fig. 2). Now that the MLA was more than 2.8mm^2^, with its’ reference vessel diameter more than 3 mm, stent was not needed according to the guidelines of IVUS. However combined with the classical symptom and ECG changes of the patient, FFR was performed further. The result of distal segment of LAD was 0.31 at rest, suggesting significant stenosis. Adenosine was then given intravenously, and the value measured for maximum myocardial congestion was 0.36 (normal > 0.8), and during adenosine administration, ECG monitoring of the patient showed ST-segment elevation of 0.2–0.6mv in leads II and III, ventricular premature (Fig. 3), and the patient complained of chest pain. Then the FFR guidewire was withdrawn and the FFR values were recorded at different parts of the lesion. The FFR values of a and b points of LAD were both 0.36 (Fig. 1b), which were positive but without pressure gradient between the two points. In the proximal and middle segment of LAD lesions, the FFR values at point c was 0.50 (Fig. 1C), and that at point d was 0.96 (Fig. 1D). The diffuse lesion was divided into two different pressure regions by the point b, c and d. The pressure gradient of c–d zone was 0.46, and the pressure gradient of b–c zone was 0.14 (normal pressure gradient is < 0.1). Although stent implantation was not recommended in the myocardial bridge area, a 3.0 × 24 mm stent was implanted in c–d zone. After full expansion, FFR test was performed again, and the values at point a and point b were both 0.89 after the maximum myocardial congestion. The pressure gradient between point c and point d was 0.07, and the pressure gradient between point b and point c was 0.01 (Fig. 4). No symptoms of chest pain and no ST segment changes of ECG monitoring were observed during the last adenosine administration. And the minimum area of the stent is 8.67mm^2^, with good sticking and expansion, and the stent was well expanded and apposed, without coronary dissection at both ends of the stent, which was showed by IVUS after a stent implantation (Fig. 5). The ST-segment in ECG of the patient returned to normal after the operation. The patient was followed up to now for more than 10 months, and fortunately his daily life and work were returned normal.

## Discussion

The diagnosis of this patient with UAP was clear and coronary was performed. The angiography showed diffuse lesion in the near middle segment of LAD. The lesion had 3 characteristics: (1) The lesion with stenosis of 60–70% was intermediate lesion (50–90% stenosis), and does not need stent implantation according to the results of IVUS. (2) The patient was diagnosed of UAP with classical symptom, FFR was performed further which suggested of stent implantation. (3) The lesion was diffuse and accompanied by myocardial bridge. As is known to all, IVUS can indicate the nature and composition of lesion plaques in addition to providing lesion area and length. However, the case did not meet the criteria for stent implantation on these indicators. FFR guidance for PCI in patients with UAP can define the culprit vessels and improve prognosis, especially in patients with multi-vessel, diffuse lesions [1]. For this case, stent implantation was needed according to FFR. Why the strategy according to endovascular imaging and functional test were contradictory? Maybe because of the following: (1) The lesion was not significant stenosis according to endovascular imaging, but it was diffuse with the length of more than 40 mm, which could result in insufficient blood supply to the distal LAD. (2) The lesion was wrapped with myocardial bridge which could influence the hemodynamics of coronary artery, and that could be detected by functional test such as FFR.

In terms of this kind of intermediate lesion accompanied with myocardial bridge, IVUS and FFR indicated different needs of stent implantation. How to make the best choice or which is more appropriate for reference, endovascular imaging or functional test? In this case, considering (1) the patient was clearly diagnosed with UAP and FFR < 0.7, indicating he had high risk of recurrent myocardial infarction; (2) ST-segment elevation, patient’s complain of chest pain and hemodynamics changes were of significance during adenosine administration, and ECG indicated LAD was the culprit lesion. We finally decided to implant stent in the LAD lesion.

Then how does stent be implanted, and how many stents does the lesion need? For such lesion, the most direct way is to implant 2 stents from the normal distal segment to the normal proximal segment of the lesion. However, from the perspective of long-term prognosis, the effect of stents implantation at the myocardial bridge is not good [2], but if the stents aren’t implanted completely, residual angina attack may still occur. Therefore, the strategy of stent implantation is very important and tricky. Endoluminal imaging does provide information on how to avoid the bridge area, but it does not answer the question of whether to avoid it [3, 4].

Therefore, by testing the FFR values in different regions, we planed to solve the lesions with the largest pressure gradient first, and then to perform the FFR again, paying special attention to the pressure gradient change in the region across the myocardial bridge. If the pressure gradience was still meaningful, stents could be implanted; if it was meaningless, stent could be omitted. Along this train of thought, first we confirmed the culprit lesions, second we detected the significant pressure gradience was 0.46 in the lesion with myocardial bridge, then stent was implanted in this area. The FFR value of the distal LAD was 0.89, and the pressure gradience across the stent was 0.07. The pressure gradience in the distal segment of the lesion decreased from 0.14 preoperatively to 0.01 postoperatively. The treatment was satisfactory as expected. It suggested that the pressure gradience of lesions wrapped with myocardial bridge was greatly affected by the proximal segment, intervention strategy such as stent implantation should be carefully evaluated and cautiously performed.

And the patient was followed up to now for more than 10 months, and fortunately his daily life and work were returned normal, just as a systematic analysis with a follow-up of more than 9 months showed, the use of FFR guided PCI is associated with lower rates of MI and MACE rates [5].

## Conclusion

Based on this case, we conclude that FFR functional tests are complimentary to intravascular imaging tests for the coronary intermediate lesion, especially the lesion wrapped with myocardial Bridges, both in assessing the lesion and in guiding treatment.

## Data Availability

The datasets used and/or analysed during the current study are available from the corresponding author on reasonable request.

## Abbreviations

UAP: Unstable angina pectoris
LAD: Left anterior descending
FFR: Flow reserve fraction
IVUS: Intravenous ultrasound
ECG: Electrocardiogram
LVEDD: Left ventricular end-diastolic dimension
LVEF: Left ventricular ejection fractions
MLA: Minimum lumen cross-sectional area
